# The thalamo-cortical complex network correlates of chronic pain

**DOI:** 10.1038/srep34763

**Published:** 2016-10-13

**Authors:** Antonio G. Zippo, Maurizio Valente, Gian Carlo Caramenti, Gabriele E. M. Biella

**Affiliations:** 1 Institute of Molecular Bioimaging and Physiology, Consiglio Nazionale delle Ricerche, Segrate (Milan), Italy

## Abstract

Chronic pain (CP) is a condition with a large repertory of clinical signs and symptoms with diverse expressions. Though widely analyzed, an appraisal at the level of single neuron and neuronal networks in CP is however missing. The present research proposes an empirical and theoretic framework which identifies a complex network correlate nested in the somatosensory thalamocortical (TC) circuit in diverse CP models. *In vivo* simultaneous extracellular neuronal electrophysiological high-density recordings have been performed from the TC circuit in rats. Wide functional network statistics neatly discriminated CP from control animals identifying collective dynamical traits. In particular, a collapsed functional connectivity and an altered modular architecture of the thalamocortical circuit have been evidenced. These results envisage CP as a functional connectivity disorder and give the clue for unveiling innovative therapeutic strategies.

Chronic Pain (CP) is a sensory disorder^1^,^2^ characterized by extreme variability in genesis^3^,^4^,^5^,^6^,^7^, expression and maintenance^8^,^9^,^10^,^11^,^12^ and may combine different signs and symptoms, with^13^ multiscale changes from molecular to behavioral levels^14^. In addition, unlike acute pain, CP is not necessarily sustained by inputs from the periphery and it may even be unrelated to any still ongoing source^15^,^16^,^17^. Although recent advances in pharmacotherapy gained notable results^18^,^19^,^20^, CP still remains poorly or not at all managed, a fact allegedly related to stable maladaptive conditions^21^ and altered plasticity^22^ and, more in general to a still absent reference model of CP. A low-profile but convenient view can be held, however, that CP as percept is the product of neuronal (and network) activities in the central nervous system. As a matter of facts, though some plain association has been described between chronic pain perception and neuronal discharge anomalies occasionally observed in experimental conditions^23^,^24^,^25^,^26^,^27^,^28^,^29^,^30^,^31^,^32^,^33^,^34^,^35^,^36^, the association between CP and long lasting single neuron activity disorders is missing. In addition models of neuronal metabolic costs, tend to discard the hypothesis that single neurons may run at high energy consumption regimes for long periods when not for decades^37^,^38^,^39^.

Theoretical models of distributed representation of CP in the brain, like the Neuromatrix hypothesis^40^ or the Homeostatic model^41^, and more recently the more encompassing and adaptive “pain matrix”^42^, as well as of maladaptive synaptic changes^22^,^43^ diversely propose CP as the result of integrative regional activities. On a larger scale, studies estimating the global default mode showed network disruptions in chronic pain subjects in comparison with control pain free subjects^44^. In accordance to these findings, a theoretical proposal by Apkarian and colleagues has gathered together many chronic pain conditions within a model^16^,^45^. However, no hints on the subserving neuronal dynamics in the diverse CP conditions have been given ever. Due to the low eloquence of simple single unit activities, but in the need to refer to some neural context, it appears convincing to capture signs of neural expression of pain within neuronal ensembles. The supraspinal somatosensory axis and more precisely the sensory thalamo-cortical loop appears to be an ideal candidate to be explored both because its long-established involvement in sensory processes and because it ubiquitously entangles the whole sensory space. Its large scale representation, as for all complex brain networks, requires, however, a suitable set of analyses as provided by graph-theoretical tools^46^,^47^. Such frameworks can assess dynamical hallmarks describing modal aspects of varying neuronal dependences gathered in complex networks of interacting neurons^48^,^49^,^50^,^51^ also capturing pathological stigmas^52^. We thus conjugated the electrophysiological exploration of the thalamocortical circuit (TC) loop at the neuronal level with the theoretical apparatus originated by graph theory and complex network theory. To this purpose, we performed, by means of microelectrode matrices, simultaneous electrophysiological recordings from the ventral-posterolateral thalamic nuclei (VPL) and the primary somatosensory cortex (S1). We used control animals as well as three common experimental models of CP in rodents (Chronic Sciatic Ligature^53^, SL or Seltzer model, Chronic Constriction Injury, SC or Bennett-Xie model^54^ and the Peripheral Inflammation^55^, PI) evaluating the functional connections and computing sets of topological measures on the resulting graphs from both spiking activity and the Local Field Potentials (LFP). We show an accurate and significant recognition of CP rats from controls and overall measures grouping CP models within one common feature space.

## Results

To test the working hypothesis that chronic pain (CP) can produce impairments on the information processing capabilities, we recorded the neural activity by means of two 3 × 3 microelectrode matrices in four groups of experimental animals simultaneously recording the neuronal activities of the ventrobasal complex of the thalamus and the somatosensory cortex. We used a total of 52 Sprague-Dawley rats grouped as follow: 12 for the Seltzer model (SL), 12 for the Bennett-Xie model (SC), 14 for the peripheral inflammatory model (PI) and 14 for control (CR). Each recording session in the course of an experimental run lasted 1–2 minutes and the spike timings of each putative neuron were extracted by means of a spike sorting procedures. By filtering raw data onto low frequencies (1–100 Hz) we also analyzed the Local Field Potentials (LFP). Spike trains were thus binarized (choosing time bins of 1 millisecond) and split into windows of 100 ms lengths reflecting the duration of most thalamocortical interactions^49^. In such windows we assessed the extent of functional interactions separately for spiking and LFP signals, and subsequently we extracted a set of coherent complex network features.

**Figure 1:**
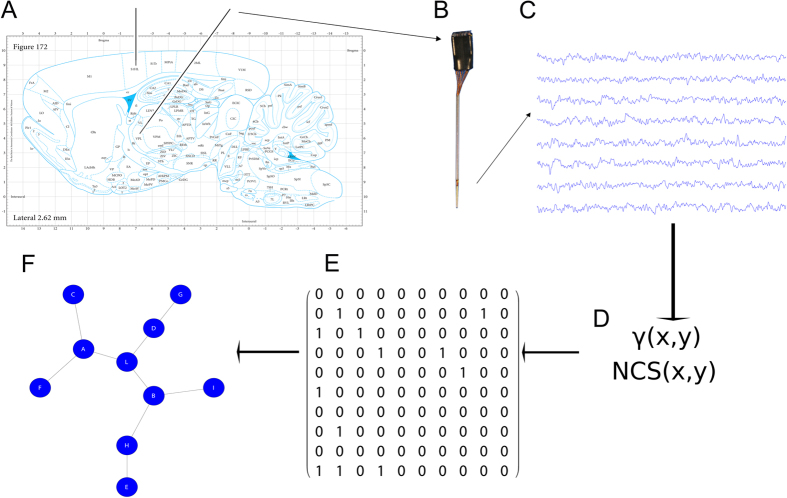
Explanation of the experimental setup. (**A**) The illustration of the stereotactile coordinate in the Paxinos’s atlas (reprint permission n. 1982084; Paxinos, G., Watson, C. *The Rat Brain in Stereotaxic Coordinates (Third Edition*), 172 (Elsevier, 2006)). The two black lines represent the electrode matrices illustrated in (**B**). (**C**) A typical time series of the raw extracellular recordings where the functional connections were extracted by means of the functions in (**D**). (**E**) A sample of a binary matrix extracted by recordings that corresponds to the graph in (**F**).

Preliminary, we wondered if fundamental features of neural activity could be predictive of the animal conditions. In particular, we analyzed the firing rate and the cross-correlation, of S1 and VPL neurons, seeking for potential discriminations between the CP class (CP = {SL, SC, PI}) and the control group. By following this track, we used a recent proposed complex mathematical framework to assess functional connections in the thalamocortical extracellular activity^49^. In the same way, on the graphs constituted by the sets of the inferred functional connections (separately spiking activity and LFP), we applied a group of common topological measures contained in a general purpose framework. The entire framework is illustrated in Fig. 1.

**Figure 2:**
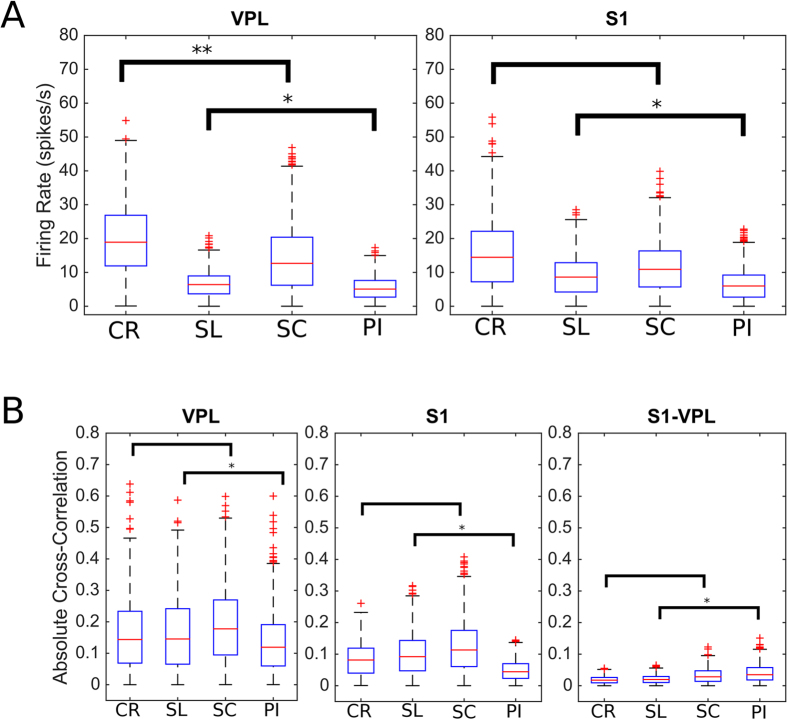
Basilar features of the spiking activity. (**A**) The plots show the distribution of firing rate (spike/s) for the recorded neurons of VPL thalamus (left) and for the S1 cortex (right). While in VPL there is a statistical significance between the normal and neuropathic animals, in S1 there is only significance among the neuropathic classes. (**B**) The cross-correlation estimated between the neurons from VPL (left), S1 (center) and VPL versus S1 (right). Cross-correlation is unable to discriminate control from neuropathic rats. CR represents the control, PI the peripheral injury, the SC the sciatic constriction (Bennet-Xie model) and the SL (Seltzer model) animals. Distributions are visualized as *boxplot* according to the standard parameters.

**Figure 3:**
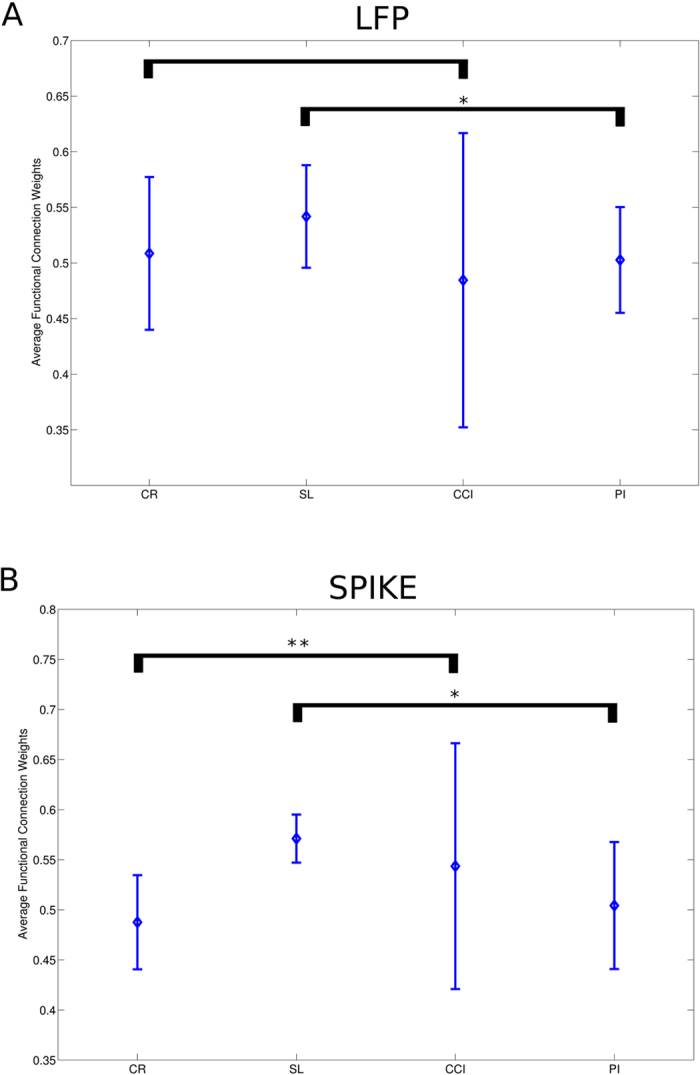
Comparison among the experimental conditions. (**A**) Average graph weights (obtained from LFP) did not statistically differ from normal to neuropathic models. Similarly, graph weights from spiking activity produce the same result. CR represents the control, PI the peripheral injury, the SC the sciatic constriction (Bennet-Xie model) and the SL (Seltzer model) animals. Lines outgoing the central balls indicate the standard deviation.

Prior to apply complex network statistics on the extracted graphs, we simply compared the distribution of functional weights in the four groups both from spiking activity and from LFPs. We found a diverging result between spiking activity and LFPs, where spiking activity (P = 0.0423, N = 15593, ranksum test, Fig. 2) did show significant differences between the CP models and the control group, differences not relevant for LFPs (P = 0.5701, N = 2340, ranksum test). Furthermore, the graph weight distributions in CP models were statistically different both in spiking activity (P < 0.000, N = 11495, Kruskal-Wallis test, Fig. 3) and in LFP (P < 0.000, N = 1710, Kruskal-Wallis test). These results anticipated that functional graphs conveyed a dynamic thalamocortical characterization, which profoundly discriminated CP from CR groups.

**Figure 4:**
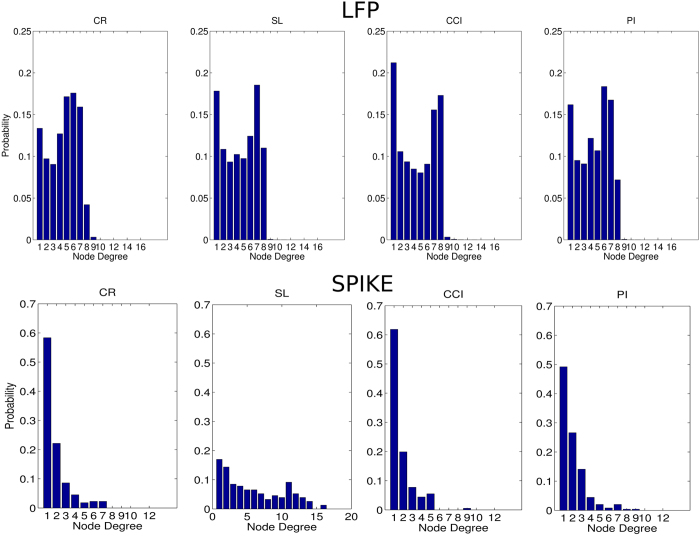
Node degree distribution along the experimental conditions and recorded signal types. The node degree histograms of the graphs obtained from the LFP (above) and spiking activity (below). CR represents the control, PI the peripheral injury, the SC the sciatic constriction (Bennet-Xie model) and the SL (Seltzer model) animals.

By analyzing all functional thalamocortical graphs, we computed the average node degree distributions (Fig. 4) and found another fundamental characterization because the significantly different node degree distributions between CP and CR animals (spiking activity: P < 0.000, N = 15593; LFP: P < 0.000, N = 2340, Kruskal-Wallis test) as well as among CP models (spiking activity: P < 0.000, N = 11495; LFP: P < 0.000, N = 1710, Kruskal-Wallis test).

Subsequently, on the functional graphs the clustering coefficient (C, the node tendency to form highly dense clusters) and the characteristic path length (L, the average shortest path length among nodes) were first estimated. These represent respectively the functional counterparts of two crucial aspects of the information processing: the former indicates the tendency of the network to form clusters of nodes (functional segregation) and the latter expresses the aptitude of the network to easily transfer information among nodes (functional integration).

In spiking activity and LFP functional graphs, we found that C and L values were significantly different between CP and CR animals (Table 1). In particular, the C values were higher in CR than in CP animals (spiking activity: P < 0.000, N = 15593; LFP: P < 0.000, N = 2340, ranksum tests) and the L values were lower in CR than in CP animals (spiking activity: P < 0.000, N = 15593; LFP: P < 0.000, N = 2340, ranksum tests). These results indicated that thalamocortical circuits of CP animals segregated and integrated information worse in comparison to normal animals. Typically, the network analyses from multiple networks compare these metrics to those computed on randomized versions of the original networks (representing null networks) toward a better comparability of the network statistics. In this work, we used a standard randomization procedure that keeps unchanged the node degree distributions to estimate the average values of σ (C/Cr) and of λ (L/Lr), being Cr and Lr the clustering coefficient and the characteristic path length computed on the randomized graph versions.

Even by considering normalizations, the σ values were higher in CR than in CP animals (spiking activity: P = 0.002, N = 15593; LFP: P < 0.000, N = 2340, ranksum tests) as well as for λ values (spiking activity: P < 0.000, N = 15593; LFP: P < 0.000, N = 2340, ranksum tests).

Furthermore, the ratio between sigma and lambda expresses an emergent property called small-worldness which measures how much a network can be considered a small-world network. Small-world networks are optimal network structures ubiquitously found in “normal” or control brain networks but lost in neurological and psychiatric disorders (such as epilepsy, Alzheimer related diseases, schizophrenia, etc.)^56^,^57^,^58^. For these reasons, since the average cluster coefficient and path length were significantly altered in CP, we expected that the functional graphs of the thalamocortical circuits of CP animals were not organized in small-world network. Indeed, we found that the index of small-worldness (S) connoted CR spiking activity functional graphs as small-world networks, but classified CP functional graphs as non-small-world networks. Instead, LFP denoted all graphs as small-world networks but with different magnitudes: CR and PI showed the highest values while SL and CCI showed the lowest values indicating that network damages were worsened, a crucial functional hallmark of the thalamocortical axis. Remarkably, this represents the fundamental result of the work because we found that S can be a coherent discriminator of the pain condition in the thalamocortical circuit of rats and configure CP as a functional thalamocortical connection disorder at the neuronal level.

**Figure 5:**
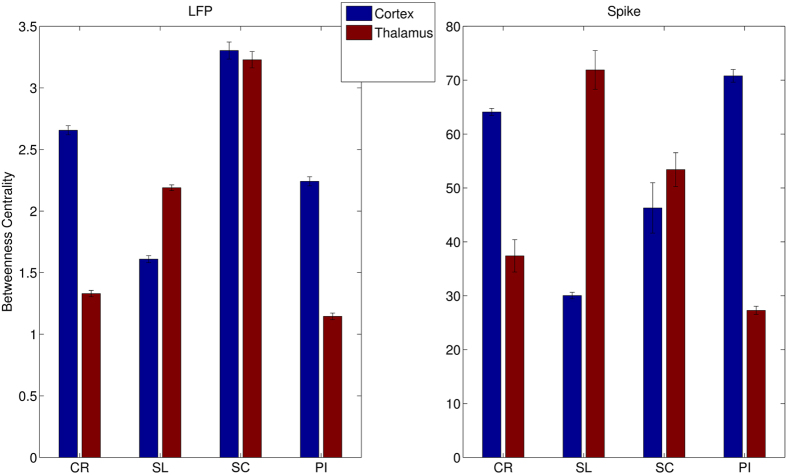
Analysis of betweenness centrality (BC). In the LFP (left), the BC is mainly distributed in S1 in normal animals while in SL the balancing is reversed, in SC appears equidistributed an in PI is barely maintained. A similar trend is shown also in the spiking activity (right). CR represents the control, PI the peripheral injury, the SC the sciatic constriction (Bennet-Xie model) and the SL (Seltzer model) animals.

Subsequently, we decided to investigate another important topological feature such as the centrality of nodes that represents a robust estimation of the load node within networks. Then, we computed where the load was concentrated along the thalamocortical axis. We found that in CR animals the network load was mostly concentrated in S1 (around 64%, Fig. 5) and the remainder in VPL nodes (36%). The load distribution along the CP models was severely altered but not coherently in all CP models. Indeed, in SL and SC models the loads were mainly concentrated in VPL but in PI models the distribution appears to be most likely to those of CP models. Hence, the centrality separated only the SC and SL models from CR rats.

**Figure 6:**
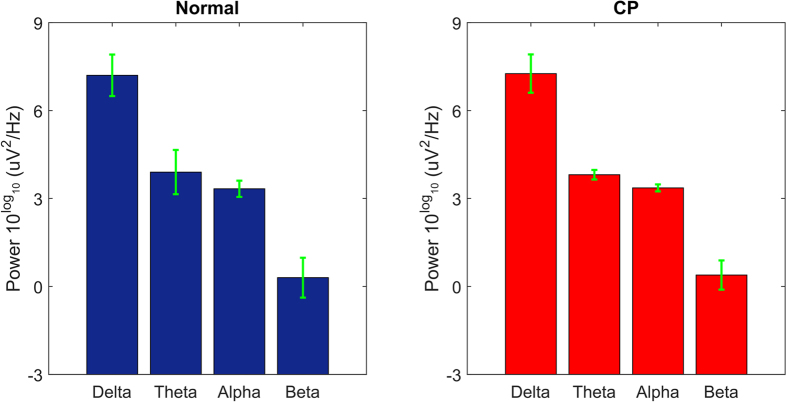
A comparison between the power spectral distributions of the four EEG electrodes recorded from normal rats (left side) and CP rats (right side). Frequencies are divided into four EEG bands: Delta (1–4 Hz), Theta (4–8 Hz), Alpha (8–12 Hz), Beta (13–25 Hz). The statistical analysis did not reveal any significant difference between the whole two distributions (P = 0.320, two-sample Kolmogorov-Smirnov test) and, by compare pair-wisely, each band (P > 0.1 in all ranksum tests).

As last assessment, we wondered if the observed CP thalamocortical functional alterations reflect significant changes in the recorded electroencephalographical activity. A positive answer likely suggested an involvement of further cortical and subcortical circuits. Oppositely, by comparing the EEG power spectral bands of the two groups we did not found any significant changes between normal and CP groups (Delta: P = 0.989; Theta: P = 0.999; Alpha: P = 0.940; Beta: P = 0.999; N = 52; ranksum tests, Fig. 6).

In conclusion, we found a set of topological features (C, L, S) able to separate thalamocortical functional graphs among different experimental models of CP. Notably, these results indicate that chronic pain can be configured as a functional connectivity disorder. In this new perspective, novel interpretations and potential treatments for chronic pain should emerge.

## Discussion

In this work we were able to extract common anomalous thalamo-cortical network signs in experimental CP models with different aetiology and pathogenesis. More in detail, common topological features highlighting the presence of CP have been detected and well identified by graph theoretic measures in the thalamo-cortical network. We explored specifically the thalamocortical axis because its prominent role in sensory input processing, aside from the many other brain regions committed to in pain processing^59^.

Many previous works on chronic pain were indeed focused on the thalamocortical recurrent networks in a number of pathological states, CP included. For instance, high thalamocortical theta coherences have been shown in patients with chronic neurogenic pain^60^. In earlier observations, Llinas and co-workers^61^ showed abnormal, internally generated low frequency oscillations in the thalamocortical circuit interfering with the normal state-dependent flow of information between thalamus and cortex in subjects with chronic pain. These prominent studies carried out with imaging or electrocorticographic techniques evidenced global or regional changes in network dynamics surfacing in chronic pain states. No study has however carried out on lower scales taking into account collections of single neurons lying in different regions of the brain and analysing them in terms of connectivity and graph architectures. As from the literature, possible candidates to might have already been identified, such as cellular memory processes^34^. These could, indeed, contribute a reasonable support to the hypothesis, being observable in many CP models such as LTP like processes in the spinal cord or the synthesis of specific related molecules (such as isoforms of protein kinase C PKMζ^62^), that underlie long-term memory storage in various brain regions^8^,^63^.

However, memory processes are not ubiquitary identical in the various stations of the nervous system^64^,^65^ and this weakens the instance of a common memory-related model. Thus, because neither the molecular nor the large neuron populations’ levels appear to satisfy the tentative appraisal of a stable reference to the common substrate of the percept of pain, it looks judicious to start from a mere neuron level, yet with relevant cautions.

Original studies on single neuron recordings showed consistent analogies among typical chronic pain clinical signs (such as spontaneous pain, allodynia and hyperalgesia) and characteristic neuronal discharges (such as spontaneous neuronal hyperactivity and hyperresponsiveness to non-noxious or noxious stimuli). As already noted, however, an assumption of “single neuron pain coding”, namely of capability of single neurons to code for pain may be better held in acute states, when noxious inputs generate discharge profiles that appear complementary to intensity and duration of peripheral stimuli. At last, single neurons do not appear to express univocally the different substrates of chronic pain but sparsely, namely only a fraction of the recorded neurons behaves along the plain model of “so much damage, so much pain, so much activity level” and for momentary conditions, apparently coherent with the condition of CP (continuous state of pain accompanied by enhanced activity levels). In fact, during the neuronal recording sessions from CP animals, neurons showing hyperactivity or hyperresponsiveness at the beginning of the recording may become unresponsive or silent in the next stages to reappear in further stages of the recording^8^,^23^,^27^,^32^,^33^,^34^. All these issues indicate that durable signs of CP at the neuronal level must be searched elsewhere. In addition, hypotheses of permanent or long lasting high rate neuronal activities meet severe flaws with neuronal biophysical limitations and metabolic data. As it has been evidenced in many studies, neurons are tuned to minimize metabolic costs subject to functional constraints^66^. In our model of CP, the parsimonious use of energy levels is also related to the connectivity costs of the strength of synchronized oscillations. This is enhanced when the wiring cost of the networks is increased above minimum by the inclusion of long-distance axonal projections, which mediate topological short-cuts between spatially remote oscillators^67^,^68^.

In the thalamo-cortical circuit the long range connections require thus an additional energetic expenditure that appears to be collapsed in the conditions of CP where the information transfer capability of the network fails as reported in theoretical and experimental works on the small-world network dynamical properties and on the consequences of edge reconfiguration^69^,^70^.

In any case, in line with the current literature showing the scarce global representation efficiency of single units in comparison with faithful prediction, encoding and decoding properties of neuronal networks^71^, we performed analyses on medium-large scale network data obtained from simultaneous thalamo-cortical neuronal recordings. With these approaches, topological representations of complex brain functional architectures have been shown highly efficient in capturing the core nature of circuitry dynamics, enabling refined trait recognition in connectivity and information transfer from different sources.

Brain topologies have recently been widely applied in studies with imaging (fMRI, PET) and in large-scale electrophysiology (high resolution EEG and MEG) techniques^47^,^72^. These topologies represented by graphs are distinguished by peculiar spatial organizations of their elements or nodes and the relative connections among the nodes^73^.

On large-scale brain networks, it has been observed that eventual disruptions of the robust balance between integration and segregation are tightly related to pathological conditions (Schizophrenia, Epilepsy, Autism, etc.). Indeed, a small-world organization appears a fundamental requirement for the proper physiology of information processing in the brain^49^,^50^,^74^ as well as in spontaneous activations of neuronal cultures^73^,^75^,^76^ that are lost in pathological samples^77^.

In this work, we show that small-world conditions have faded or are not detectable in the thalamo-cortical networks in the three CP models analyzed. We thus propose that TC networks in such states are definitely compromised resulting in a significantly worse ability to process information and less functionally segregated and integrated with smaller community structures^22^.

The hypotheses start from the inherent logic of complex integrated processes that can’t be predicted by linear reductions to simpler components at lower organization levels. In complex contexts, no precise assessment or priority can be assumed, *a posteriori*, to the concurring elements once integrated in an emergent context at higher levels of complexity. In other words, the huge number of elements concurring to express CP (such as transmitters, receptor densities, types, ionic or metabolic timing, network architectures, lose or strict spiking coincidences just to name a few) involved in the exhaustive expression of CP at higher scales can’t be equally evaluated or weighted as it is done at lower scales. According to the laws of complex systems it is possible that they may play prominent or unrecognizable roles in the novel context^78^,^79^. This addresses the inherent weakness of the large catalogue of cause-effect relationships of single events vs the global expression of CP.

An advantage of a meta-analysis to CP as it is enabled by our model allows for extensive evaluation that go beyond the boundaries of the theme. As it is widely debated, chronic pain has no apparent evolutionary or adaptive value^80^ showing tracts seemingly at odds with the “logic of living”. A recent paper on mollusks suggests that long-lasting nociceptive sensitization could have provided a darwinian adaptive trait enabling faster environmental responses and concurrent better survival rate^81^. The topological approach provides an opportunity to evaluate the neuronal dynamics from a more synthetic and general observation point. In a darwinian perspective, in CP the sensory system appears to have come to an exhaustion of plasticity resources, a state where it, assumes a minimal energy configuration. The stability of the configuration (reduced information transmission, loss of the architectural graph properties etc.) might be interpreted as a late and final strategy just to avoid the ignition of destructive local network processes in case of newly pushing environmental conditions that would burn-out the residual dynamic schemes. In support of this hypothesis, the common trait of neuronal and network collapse of information transmission, with a monotonous topological shift from small-world architectures to lattices, appears as a loss in modularity (and vice-versa). Modularity, inherent to small-worldness, is an adaptive strategy adopted by living organisms or in brain evolution to match rapidly changing environments^82^. In other words, the networks involved in CP rehearsals with anomalous stabilization have no energy to match the changing environment and are riveted to energetic still state just to avoid at most the continuous sensory requests of the external world that would act as destructive functional pressures.

From a clinical point of view, classifying chronic pain as a connectivity disorder may generate significant implications. That CP has to be rethought as an idiotype, this has been subtly sensed by some author^83^. The immediate consequences have to be thoroughly considered. From a theoretical point of view, it is proposed here for the first time a novel complex-network-based interpretation of chronic pain that can lead to potentially new modeling of chronic pain and to novel therapeutic assessments by means of topological network measures.

Due to the important consequences on the therapeutic plans of CP, it is worth to observe that the current therapeutic plans of a chronic pain control are focused on the reduction of the perceptual condition, i.e. pain. This action starts, however from an absent general appraisal of the nature of chronic pain itself. With the new theoretical frame that we are presenting here it becomes conceivable a deep and innovative reconsideration of all the therapeutic plans, requiring different strategies and definitely different drugs. Usual pain management by opioids or other drugs influences or impairs patients’ alertness, mood state, or reactiveness along with inducing concurrent organ or systemic disorders due to collateral effects. Drugs that further reduce the neuronal network efficiency and connectivity strength by dropping the overall activity rates of neurons appear thus in conflict with the tenets of our assumptions. Therefore, the requirement for a seemingly suitable therapeutic appraisal should provide an opposite strategy with the “injection” or “delivery” of information into the central circuits involved by the anomalous sensory inputs. How and how far these interventions have to be programmed, though still unknown, could be potentially envisaged in the scenario of novel technological tools such as focused electrical or optogenetic techniques^84^,^85^, coupled, for instance, to ultrasound driven localized microdisposal of stimulatory drugs^86^,^87^ able to circumvent the blood-brain barrier.

## Materials and Methods

### Ethical Statement

All the animals have been used in accordance to the Italian and European Laws on animal treatment in Scientific Research (Italian Bioethical Committee, Law Decree on the Treatment of Animals in Research, 27 Jan 1992, No. 116, guideline n. 86/609/ European Community). The National Research Council, where the experiments have been performed, adheres to the International Committee on Laboratory Animal Science (ICLAS) on behalf of the United Nations Educational, Scientific and Cultural Organizations (UNESCO), the Council for International Organizations of Medical Sciences (CIOMS) and the International Union of Biological Sciences (IUBS). The research has been approved by the Ministry of Health and classified as “Biella 1/2014” into the files of the Ethical Committee of the University of Milan.

### Electrophysiology

Fifty male albino rats (Sprague-Dawley, Charles River, Calco, LC, Italy, 270–350 g) were chosen out of the set of the animals employed in the research.

The rats underwent preliminary barbiturate anaesthesia (50 mg/kg ip) for the surgical experimental preparation. The trachea was cannulated to gain a connection to the anaesthesia-ventilation device. A 16-gauge butterfly was then positioned in the root of the lateral tail vein. A surgical opening was then done over electrodes the skull vault with the removal of the skin the *galea capitis* muscles and of their fibrous insertions over the parietal bones. The periosteum was then delicately scraped off from the parietal, frontal and occipital bones. The rats were then placed in a stereotaxic frame and the animal were paralyzed by intravenous Gallamine thriethiodide (20 mg/kg/h) injection and connected to the respiratory device delivering (1stroke/s) an Isoflurane^®^ 2.5% 0.4 to 0.8 l/min in Oxygen 0.15–0.2 l/min gaseous mixture. Curarization was maintained stable throughout the whole experiment by Gallamine refracted injections. During the experiment the anaesthesia level was continuously monitored by the EEG recordings by four leads placed, respectively in a fronto-occipital row (in stereotaxic coordinates referenced to the bregmatic point, where *F*, *P* and *O* refer to Frontal, Parietal *F* + 1.2 mm AP-2.2 mm LL; *P1*: −1.2 mm, 2.5 LL; *P2*: 3 mm AP, 2.7 mm LL; *P3*: 5.5 mm AP, 2.7 mm LL; The fifth, reference lead, was placed on the occipital bone posterior to the Lambda reference point. *O*: −2 from Lambda, 2 mm LL). Briefly, five partial bone holes have been trephined at those reference points and five external leads were then placed in the bone cradles and connected to the recording system. A hypertonic salted cream was used to ameliorate the electric interface between the leads and the bone. During the experiment the EEG recordings continuously monitored the anaesthesia level.

We simultaneously recorded spiking and local field potential activities were simultaneously recorded from anesthetized rats, by two microelectrode matrices from three stations in the brain: the thalamic ventro-postero-lateral complex nuclei (VPL) and the primary somatosensory (S1) cortex. We also concurrently recorded from four EEG derivations. The neuronal electrophysiological recordings have been obtained contralaterally to the stimulated paw; the concurrent EEG recordings were obtained ipsilaterally to the stimulated paw.

The neuronal recordings were obtained by two matrices of extracellular tungsten or Pt-Ir electrodes were framed in 3 × 3 arrays of single shanks, inter-tip distance 150–200 mm, tip impedance 0.5–1 MΩ (FHC Inc., ME, USA). The coordinates have been estimated from the Paxinos – Watson Stereotaxic Atlas of the Rat Brain^88^. In detail, the two matrices were placed at −1.2 mm AP, 2.6 mm LL and −6 mm AP, 2.5 mm LL for the somatosensory cortex and the thalamic nuclei respectively. The thalamic regions were targeted with a postero-anterior slant of 25° of the matrix to avoid spatial interferences with the cortical matrix. This obliged to recalculate the depth by a simple geometric correction of the usual measure. The cortical matrix was inserted 400 μm deep at the superior border of the granular layer and then slowly advanced, by an electronically controlled microsteppers (Narashige, Japan), at 10 μm steps for probing the responses of local neurons to exploring sensory stimuli on the contralateral posterior paw, until a clear response was evident and repetitive on at least six out of the 8 recording microelectrodes of the matrix within a final depth of 600 μm. The thalamic probe was advanced electronically by a second electronically driven microstepper (Transvertex, Stockholm) from a starting depth of 4700 μm down to 5800 μm (fastly driven to avoid tissue damage). The concurrent EEG recordings were obtained ipsilaterally to the stimulated paw.

### EEG recordings

The EEG electrodes were placed along the stereotactic coordinates (in front-back order, Bregma as relative zero AP, zero ML reference point) as follows: FrontalAnterior (FA) +3 mm MedioLateral (ML) −2 mm, ParietalAnterior (PA) −0.15 mm, ML −2.8 mm, MiddleParietal (MP) −3 mm, ML. −3 mm, PosteriorParietal (PP) −6 mm, ML. −2.8 mm, reference in Occipital bone −9 mm, ML. 2 mm. (121). For the analyses, we selected preferentially the EEG data from the second derivation placed over the sensory cortex mirroring the contralateral somatosensory primary cortex where the neuronal recordings were obtained.

### Electrophysiological extracellular recordings

For the electrophysiological recordings, two holes were drilled on the skull of 3 mm^2^ for the cortical and the thalamic matrix accesses. The holes were drilled centered respectively on the cortical access centered around a reference point at −1.5 mm AP and 2.5 mm ML, and the electrode was driven around 450 to 800 micrometer deep by an electronically controlled microsteppers (Narashige, Japan). The thalamic access hole was centered at −6 mm AP and −2.5 mm ML. The thalamic matrix was inserted with a slant at 25° and driven at least at 5500 micrometers in depth and then advanced electronically by a second electronically driven microstepper (Transvertex, Stockholm) until full responses were observed to peripheral test stimuli. Fast thalamic and cortical responses to light tactile stimuli with a brush-test on the sciatic innervation field (the plantar aspect of the left hindlimb) were the anatomo-functional acceptance criteria for acquisition. All the experimental blocks were organized with periods of ongoing activity recordings lasting around 20 min. and not more to preserve at most the data homoscedasticity, in the additional stable conditions of gaseous anesthesia. After a cycle of spontaneous and stimulated activities was completed, we repeated twice the original recordings. Then we advanced in depth the electrodes, 20 mm and 100 mm respectively for the cortical probe and the thalamic matrix ensemble, to reach an adjacent region, then checking again with the test stimulus the responsiveness of the newly recorded regions. In positive cases we repeated the recording cycle as above. We recorded from five to six stations in progressive steps for each animal.

For signal amplification and data recordings a 32 channel Cheetah Data Acquisition Hardware was used (Neuralynx, MT, USA, sampling frequency 32 kHz). Electrophysiological signals were digitized and recorded with bandpass at 6 kHz and 300 Hz for spikes and at 475 Hz–1 Hz for the EEG. The data stored were analysed off-line both by Matlab and by locally developed software. A histological confirmation of the placement of the electrodes was then obtained on brain coronal sections stained with cresyl-violet.

### Preliminary data analyses

For signal amplification and data recordings a 40 channel Cheetah Data Acquisition Hardware was used (Neuralynx, MT, USA, sampling frequency 32 kHz). Electrophysiological signals were digitized and recorded with bandpasses at 6 kHz/300 Hz for spikes, 180 Hz/1 Hz for Local Field Potentials. The data stored were analyzed off-line both using Matlab and by locally developed software. The neural firing rates had a mean of 31.4 Hz with standard deviation of 26.8 Hz.

After the recordings the LFPs were downsampled to 0.5 KHz. We used for filtering the same techniques described in. After filtering and downsampling, the spike contamination of LFP signals was null avoiding further spike removal techniques. The spikes were extracted and sorted by using the *Wave_clus* MATLAB toolbox. Sorted cells with average rates below 4 Hz and above 100 Hz were excluded from the analysis. Furthermore, neurons resulted from sorting which had improbable inter-spike-interval distributions were discarded as well. Recorded neurons were uniformly distributed over the recording matrices and every electrode show distinct neural activity otherwise the matrix was repositioned. At the end of this process, we collected a total of 391 neurons (56 ± 17 in each experiment) out from the set of the acquired signals.

The timestamps of spike occurrences were represented by binary sequences where 1’s labeled a spike. We considered time bins of 1 ms thus avoiding occurrence of multiple spikes within the same bin.

Finally, we split each sequence into fixed-length (100 ms) overlapping windows (Fig. 1), thus obtaining an ordered set of equal length windows.

In order to discriminate groups of recording by the firing rates, we verified the intraclass consistency of the recorded spiking activity within each experimental condition. To this purpose we performed a one-way analysis of variance on the four sets of experiments. No significant difference within each class was observed.

### Functional connections by spike-train similarities

Interactions between neurons can generate very complex, time-delayed and asymmetric patterns especially in the thalamocortical circuitry. In this work, we reproposed a framework successfully applied in a similar context wherein Markov stochastic models model spike trains^89^.

We used the function Normalized Compression Similarity (NCS), formally defined as: given that x and y are two spike trains, the NCS is equal to:


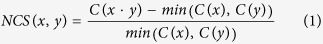


where the C function represents the compressed sequence length and is the sequence concatenation operator (e.g. 0101 · 101 = 0101101). If *NCS*(*x*, *y*) is close to 1, the sequences *x* and *y* are considered similar. If close to 0, the sequences are strongly dissimilar.

We evaluated the NCS function on time windows (100 ms length) of the recorded spiking activity assuming that relative high values of similarity corresponded to actual functional connections (Fig. 1E).

### Functional connections by LFP phase synchrony

LFPs are low frequency signals reflecting a wide range of synaptic events. In this work we investigated the synchrony of LFP phases originated in different recording sites during spontaneous and tactile evoked activities. We measured phase synchronies between two recorded LFP sequences (x and y) by the following function


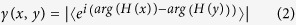


where *e* is Napier’s constant, H is Hilbert Transform, *arg* is the argument function and *i* is the imaginary unit^90^. The Hilbert transform and the argument were computed with, respectively, the *hilbert* and the *angle* Matlab functions. When *γ (x*, *y*) is equal to 1 (0), then x and y are perfectly synchronous (asynchronous).

### Complex Brain Network

By using the NCS and *γ* functions, we estimated the functional connections of the recorded neuronal networks. We first split each recorded sequence into 100 ms time windows (Fig. 1) and then we computed the adjacency matrix for all neurons or electrodes. The resulting matrices exhibited values in the unitary interval. The functional connections extracted from extracellular recordings are the counterpart of non-oriented graphs.

Since in previous work we noticed that variable thresholds equal to a higher percentile of the weight distribution and vary between 0.2 and 0.8, did not affect result we selected the 75 percentile.

For the analysis of these graphs, we introduced a set of common statistics from the Complex Network Theory able to detect possible matches between the extracted graphs and prominent topologies like small-world networks (Table 1)^91^,^92^. A small-world network is generally obtained by evolving a basic ring lattice graph, where each node is connected to their K neighbours. The chosen neighbourhood involves typically much less nodes than the total node number N. Randomly adding and removing edges from the starting graph achieve the graph evolution. The resulting graph has many, typically small, quasi-complete subgraphs (cliques) where each node is connected to every other node in the clique. Furthermore, small-world networks exhibit short average distances between nodes^46^,^47^.

From a functional perspective, small-world networks can express two important information-processing features: information integration and segregation. Functional segregation recruits specialized processing within densely interconnected nodes (cliques). Functional integration combines information processed in distributed nodes or cliques. These network properties can be measured by two statistics: the clustering coefficient (C) and the characteristic path length (L). The former measures how close the neighbours of a node are to being a clique. The latter estimates the average shortest path length in the graph, i.e. how much the nodes are accessible. Both measures, implemented in a Matlab toolbox, were used for our network analyses (clustering_coef_bu.m, charpath.m)^93^.

In complex network theory, several graph measures take specific meaning only if they are compared to the same graphs subject to randomization or latticization (often called *null networks*). Both procedures guarantee that the node degree distributions of the original graphs were preserved. We computed, by using the Matlab function randmio_und.m, the randomized version of our graphs and we estimated C^r^ and L^r^. These null network values are required to verify the small-world nature of the graphs.

The functional graphs obtained by our analysis were further characterized to study the information flow. For this aim, we computed a measure of centrality (betweenness) for graph nodes, an estimate of the number of shortest paths from all vertices to all others that pass through that node. Because it can be interpreted as a measure of the load of a node within the network, the distribution of node centrality highlights how the information flow is balanced within graphs.

Ultimately, we analyzed networks that evolved in time dropping and recruiting nodes and connections and networks from different experimental conditions. Such a methodology requires the discussion of potential issues.

First, unconnected nodes were rare but could occur after adjacency matrices were binarized. For this reason, we removed graphs in which less than the 99% of nodes were connected.

**Figure 7:**
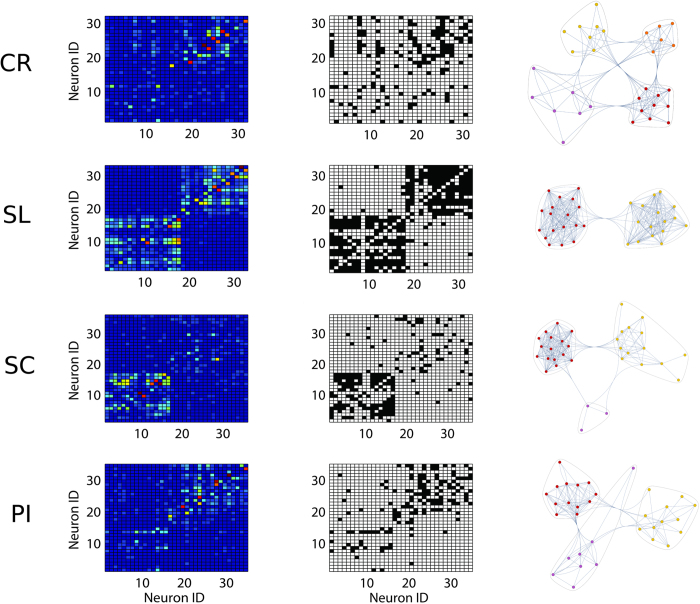
Samples of the spiking activity functional graphs for each experimental condition. First column reports the raw weighted adjacency matrices while the second column indicates the respective binarized versions. The third column represents the graphs as plotted by the “CommunityGraphPlot” function (Mathematica, http://www.wolfram.com). Remarkable differences are appreciable both among CP conditions and between CR and CP condition. CR represents the control, PI the peripheral injury, the SC the sciatic constriction (Bennet-Xie model) and the SL (Seltzer model) animals.

Second, network statistics were applied on network with different sizes (for spiking activity) because the recording sessions returned a variable number of active neurons. However, by analyzing the observed variance of network size we concluded that C and L couldn’t be significantly affected by our network size changes. Significant changes appeared for synthetic networks that increased their size by orders of magnitude. However, we discarded graphs that were outliers (beyond 5^th^ and 95^th^ percentile) of the node, edge and density number distributions in order to obtain a better homogeneity^49^. In the work, we refer to these two conditions as *admissibility criteria*. Samples of functional graphs (represented by their weighted and binarized adjacency matrices) from different groups are shown in Fig. 7.

## Additional Information

**How to cite this article**: Zippo, A. G. *et al.* The thalamo-cortical complex network correlates of chronic pain. *Sci. Rep.* **6**, 34763; doi: 10.1038/srep34763 (2016).

## Figures and Tables

**Table 1:** Complex network statistics commonly used in this work.

Measure	Definition	Interpretation
Node degree	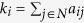	Number of edges connected to a given node *i*. Nodes with relatively high values of *k* are called *hubs*
Shortest path length	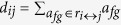 where *r*_*i*↔*j*_ is the shortest path between *i* and *j*	The number of edges encountered in the shortest path between node *i* and *j*
Characteristic path length	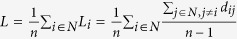	Measure of network integration. Small values identify strongly integrated networks
Clustering coefficient	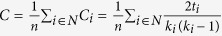 , with 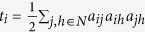	Measure of fine-grain network segregation. It counts the average number of triangles *t* (3-node fully connected graphs) present in the network
Modularity	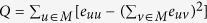 , where *M* is a partition of *V* (whose elements are called *modules*) and *e*_*uv*_ is the proportion of links that connect nodes in module *u* with nodes in module *v*	It evaluates the tendency of the network to be reduced in independent (or scarcely dependent) modules
Betweenness Centrality	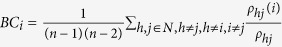 , where *ρ*_*hj*_ is the number of shortest paths between h and j, and *ρ*_*hj*_(*i*) is the number of shortest paths between *h* and *j* that pass through *i*	It is the amount of shortest paths that pass through the node *i*. It roughly indicates how much information burdens the node *i*
Small-worldness	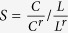 , where *C*^*r*^ and *L*^*r*^ are the randomized version of the original network; *S* >1 denotes small-world networks	It determines how much the network is a small-world network.

All formulas are referred to a (undirected) graph 〈*V*, *E*〉, with |*V*| = *N*, opportunely described by the adjacency *N* × *N*-matrix *A* = *a*_*ij*_ where *a*_*ij*_ = 1 if and only if there exist the element (*i*, *j*) in the set *E* and 0 otherwise.
